# Making health insurance pro-poor: evidence from a household panel in rural China

**DOI:** 10.1186/s12913-015-0871-7

**Published:** 2015-05-29

**Authors:** Mateusz J. Filipski, Yumei Zhang, Kevin Z. Chen

**Affiliations:** Development Strategy and Governance Division, International Food Policy Research Institute, 2033 K street NW, Washington, DC 20815 USA; Agricultural Information Institute - Chinese Academy of Agricultural Sciences, Key Laboratory of Agri-information Services Technology, Ministry of Agriculture of China, No.12 Zhongguancun Nandajie, Haidian District Beijing, 100081 China

**Keywords:** NCMS, Reimbursement, Medical inequality, Public policy

## Abstract

**Background:**

In 2002, China launched the largest public health insurance scheme in the world, the New Cooperative Medical Scheme (NCMS). It is intended to enable rural populations to access health care services, and to curb medical impoverishment. Whether the scheme can reach its equity goals depends on how it is used, and by whom. Our goal is to shed light on whether and how income levels affect the ability of members to reap insurance benefits.

**Methods:**

We exploit primary panel data consisting of a complete census (over 3500 individuals) in three villages in Puding County, Guizhou province, collected in 2004, 2006, 2009 and 2011. Data was collected during in-person interviews with household member(s). The data include yearly gross and net medical expenses for all individuals, and socio-economic information. We apply probit, ordinary least squares, and tobit multivariate regression analyses to the three waves in which NCMS was active (2006, 2009 and 2011). Explained variables include obtainment, levels and rates of NCMS reimbursement. Household income is the main explanatory variable, with household- and individual-level controls. We restrict samples to rule out self-selection, and exploit the 2009 NCMS reform to highlight equity-enhancing features of insurance.

**Results:**

Prior to 2009 reforms, higher income in our sample was statistically significantly related to higher probability of obtaining reimbursement, as well as higher levels and rates of reimbursement. These relations all disappear after the reform, suggesting lower-income households were better able to reap insurance benefits after the scheme was reformed. Regression results suggest this is partly explained by reimbursement for chronic diseases.

**Conclusions:**

The post-reform NCMS distributed benefits more equitably in our study area. Making health insurance pro-poor may require a focus on outpatient costs, credit constraints and chronic diseases, rather than catastrophic illnesses.

## Background

Public health insurance schemes often have pro-poor motives. Among their stated goals is to help provide health services to those who cannot afford them. Justifications for this range from the ethical (access to healthcare is on the United Nations list of basic human rights), to the epidemiological (preventing outbreaks requires keeping everyone healthy) to the economic (a healthy labor force is more productive). Yet designing a health insurance scheme that is pro-poor is not straightforward, and success in that respect depends on how the scheme is used, and by whom. If the wealthy benefit more than the poor from an insurance scheme, then public funds are perhaps not being used optimally, or at least not consistently with the scheme’s intentions. To make health insurance pro-poor, it is crucial to understand what kind of scheme allows the poor to reap the healthcare benefits they need. This paper sheds some light on these questions in the context of rural China, by analyzing the relationship between incomes and healthcare expenditure reimbursements.

The interactions between poverty and health are pervasive and complex [1], and much attention has been given to documenting and measuring health inequity [2]. Economists have studied income-related inequality in health outcomes across countries [3] or within populations [4–6]; income-related inequality in the use of healthcare services [7, 8] or healthcare expenditures [9–11]. The inequalities they highlighted provide much of the justification for pro-poor public health insurance schemes.

Work on the relationships between poverty and health insurance has usually focused on one of two questions: whether insurance allows the poor to access health services [12, 13], and whether it prevents medical impoverishment [14–16]. Much of this literature uses identification strategies based enrollment into insurance schemes [17–19], or on the size of user fees [20, 21]. Such work needs to overcome a number of econometric hurdles [22] that can cast doubt on the validity of estimates, such as the fact that less healthy individuals may self-select into insurance schemes, that the rich and the poor may have different types of health issues, or that the cost of health services may vary with demand. If health issues vary with income, the use of specific healthcare services may not be a good measure of how pro-poor an insurance scheme is.

We avoid many of those pitfalls by focusing on the relationship between incomes and healthcare reimbursements. Reimbursements can be seen as a measure of how “pro-poor” the policy is, irrespectively of issues of endogenous healthcare needs and costs. A “pro-poor” policy would be designed and targeted in a way that already accounts for those issues, so as to provide the poor with reimbursements. We can thus ask the simple question of whether access to reimbursement is income dependent. We use a unique dataset from China to answer this question.

China runs the world’s largest network of basic medical insurance, the New Rural Cooperative Medical Scheme (NCMS). The NCMS offers a unique opportunity to study health insurance schemes because, where it exists, it is virtually universal and exclusive: we need not worry about self-selection or about payments from private insurance schemes. The question is not so much whether the poor can get insurance coverage, but rather whether they are able to benefit from what their health insurance has to offer. Launched in 2002, NCMS was gradually expanded to cover almost all rural areas today.^1^ We use panel data from three villages in Puding county, Guizhou province, to test whether NCMS is meeting its equity goals. We take advantage of program reforms that took place in 2009 to investigate the ingredients of pro-poor health insurance.

### NCMS and its reforms

The government began NCMS trials in select counties in 2002. The program grew rapidly. From 40 million participants in 2004 [23], the number of enrolled reached about 832 million by 2011, with a 97 % enrollment rate [24]. NCMS is jointly financed by governments and participants. The share of government subsidy was about 75 % in total NCMS funding in 2011.

In the early years of the scheme, coverage was limited to inpatient services, with the goal of relieving the burden of catastrophic illnesses and accidents [25]. Reimbursement rates were relatively low, and the reimbursement process was discouragingly complex [26]. Governments continued to reform NCMS year by year, increasing both the breadth of coverage (types of reimbursable expenses) and the depth of coverage (reimbursement rates and ceilings), but also raising premiums and subsidy costs to central and local governments. The average government subsidy for NCMS enrollees increased tenfold since the inception of the scheme, from 20 RMB to 200 RMB per person per year; total public expenses reached 242.9 billion RMB [27]. The insurance was used in 1.3 billion instances nationwide during 2011, 1.6 times per enrollee on average [28]. The program and its various reforms were all implemented gradually throughout the country, such that program timelines vary by province and even by county.

In Guizhou province in particular, NCMS trials started in 2005, followed by overall implementation in 2008. The number of rural households who participated in NCMS increased from 2.26 million in 2005 to 30.74 million in 2011, and the share of participants within program locations rose from 63 to 97 % of the population. The total premium per person (including government subsidy) was increased from 30 RMB in 2005 to 140 RMB in 2010, and 230 RMB in 2011. Only a fraction of this premium is paid by enrollees themselves: 10 RMB from 2004 to 2007, 20 RMB in 2008 and 30 RMB in 2009–2011. The government’s total NCMS expenditure in Guizhou increased from 43.6 million RMB in 2005 to 5,225 million RMB in 2011. The number of reimbursements was increased from 1.32 million instances in 2005 to 44 million instances in 2011. The average reimbursement rate for inpatient expenses was raised from 46.6 in 2008 to 56.8 % in 2011 [29].

In Puding county where our data comes from, the program started in 2006, and at first only covered inpatient services. Reimbursement of general outpatient expenses and large expenses related to select chronic diseases was implemented in 2009. Instantaneous reimbursement was also launched in that year, relieving patients from the need to front their medical costs. The year 2009 thus marks a major reform of NCMS in Puding, expanding the coverage beyond hospitalizations and implementing simultaneous reimbursement. We exploit these reforms to explore the relationships between income and NCMS benefits.

### NCMS, poverty, and inequality

Given its scale and ambitions, the NCMS has been widely studied, particularly in the national literature. Most studies of the impact of NCMS focus on access to care and on medical expenditures [19, 26, 30–34].

The few studies that consider the effects of NCSM on poverty and inequality differ in their conclusions. Xu and collaborators [35] suggest NCMS alleviated disease-related poverty using a survey of poor households in Zhejiang province. Qi [36] also suggests that the NCSM significantly decreased the poverty rate at the household and provincial levels, raised incomes for low and middle income households, and reduced inequality within villages. Tan and Zhong [37] focus, as we do, on the distribution of reimbursements, and find that low income groups received higher reimbursements from NCMS than high income groups. Conversely, other researchers show that out-of-pocket health payments remain a severe burden for rural households, and financial protection from the NCMS is limited [38]. Others suggest the NCMS suffers from poor design and inefficiencies, and fails to protect against impoverishment [25, 30].

One reason for such diverging results is that NCMS was reformed several times during its ten years of existence, with reform schedules varying from one county to the next. Therefore, the single- or two-period cross sectional methods used in existing studies can be looking at widely different versions of the NCMS in terms of coverage, premiums, and reimbursement rates. In this paper we partly reconcile the literature by identifying how the equity outcomes of NCMS change after the reform.

## Methods

### The puding panel dataset

This paper uses a census-type rural household panel with a total of four waves, of which we primarily use three in the analysis, as the baseline predates NCMS implementation. Data was collected in 2004 (pre-NCMS), and again in 2006, 2009 and 2011. The data cover three administrative villages in Puding County, Guizhou Province, Southwestern China.^2^ The three villages lie within 10 km of the county seat. This dataset has the advantage of covering every household in the area. As a complete census, this dataset is uniquely adapted to studying questions of inequality, because unlike with drawn samples we can be sure that the data captures any year-on-year changes in income distribution. The sample size is about 800 households in each year and 3500 individuals in each wave of the panel, for a total of *n =* 14469 observations (after 48 individuals were removed due to missing or corrupt data. No entire household was removed). All households are tracked in all 4 years of data, except for those who left the area, newly formed households (by former members of another household) or households who migrated into the area. Approval and funding for data collection was obtained from the Chinese National Science Foundation, without additional ethical review requirements for the collection of economic data.

The Puding panel surveys collected health status, medical expenditures, and medical insurance reimbursements for each household member of each home in the three villages. We have yearly data at the individual level, but not at the “health event” level: we know how much was spent on medical care for each person, but not how many times they saw a doctor or what treatment they received each time. We do have variables reflecting disease type and location of treatment for the most notable occurrence in the past year, and whether an individual suffers from any chronic disease. In addition, the dataset provides detailed individual demographics, employment information, household expenditures and income etc. Linking health and economic information in the survey allows us to analyze the impacts of NCMS on poverty and inequality, as well as to identify which socio-economic groups benefit from the insurance.

Table 1 reports statistics from our sample. This region is among the poorest and most unequal in China; Puding is on the government’s official list of “impoverished” counties. Villagers depend heavily on agriculture, even though land is scarce and soils are poor. Poverty is high but decreasing: using the 2004 national poverty line of 668 RMB per person per day and measures of deflated income (to 2004 RMB), the poverty rate decreased from 26 to 15.6 % between 2004 and 2011. Using the international dollar-a-day poverty line yields different poverty rates, but also points to sharply decreasing poverty.^3^ Inequality, however, rose in that same period: the Gini coefficient increased from 0.41 to 0.55.

**Table 1:** Survey summary statistics

	2004	2006	2009	2011
General demographics and statistics				
Number of households	795	817	862	900
Population (persons)	3380	3418	3698	4034
Household size(persons)^+^	4.50	4.34	4.45	4.57
Per capita net income (2004 RMB)	1403	1859	2420	3239
Poverty incidence (%)^# ^	26.2 %	31.3 %	23.2 %	15.6 %
Gini coefficient	0.41	0.52	0.57	0.55
Share of population in labor force (%)	65.2 %	62.3 %	62.4 %	63.5 %
Share of population 60 or older (%)	7.0 %	7.6 %	8.1 %	9.6 %
Number of migrants per HH(persons)^+^	0.45	0.73	0.73	0.87
Share of households with migrants (%)	36.7 %	39.3 %	43.4 %	41.9 %
Per capita net income (2004 RMB)	1403	1859	2420	3239
Health statistics				
Population share enrolled in NCMS (%)	na	82 %	98 %	95 %
NCMS premium	na	45	100	230
Of which: premium paid by enrollees (RMB)	na	10	30	30
Percent sought treatment^^ ^	na	56 %	60 %	45 %
Percent received reimbursement (of treated)	na	14 %	55 %	39 %
Percent reporting a chronic disease^^ ^	na	18 %	19 %	17 %

Puding panel.^+^: Average value. ^#^:2004 official poverty line = 668RMB per person-year. ^^^Not available or not comparable in 2004 survey

The number of households in the sample increased somewhat over the period, reaching 900 in 2011. The composition of the population also evolved somewhat, primarily due to migration of workers. The proportion of households with migrants increased from 36.7 to 41.9 %, which was accompanied by an increase in the share of elderly population rising from 7.0 to 9.6 %. Such shifts in the population are the reason why it is necessary to approach health-related questions with a regression framework that can control for them.

The NCMS was not active in this county at the time of the first survey wave in 2004. The second wave (2006) was the first year surveyed households could enroll in NCMS, with 82 % of households participating. Participation rose to 98 % and 95 % in 2009 and 2011, respectively.^4^ Premiums increased dramatically, from 45 RMB to 230 RMB, but most of this is publically sponsored, so that the share owed by farmers remains a small fraction of that (10–30 RMB). High participation rates suggest that self-selection into enrollment is not a major concern in this context. This allows us to focus on the question of how different enrollees benefit from the scheme. The table also shows that the share of population seeking treatment fluctuates between 45 % and 60 %. The percentage of those receiving reimbursement went from 14 % in 2006 to 60 % in 2009 to 39 % in 2011. These fluctuations suggest that disease patterns and prevalence are not stable from one year to the next. This is one of the reasons why we focus on the relationship between income and NCMS benefits rather than looking at specific treatments received, which are likely to be heavily influenced by fluctuating environmental factors.

### Empirical framework

We use a regression framework to assess whether income bears relation to benefits an individual reaps from NCMS. While benefits are individual, not all patients are income earners, such that it makes sense to pool income at the household level. The relation we are trying to test can be expressed in the following generic equation:

$$ benefit{s}_{i,t}=f\left({Z}_{i,t},\ {X}_{h,t},{T}_t, incom{e}_{h,t}\right) $$

Where *Z*_*i,t*_ is a vector of individual characteristics, *X*_*h,t*_ a vector of household characteristics, T_t_ a series of year dummies, and household income is expressed per capita. Year dummies capture the year-specific mean shifts. We can divide the sample into two periods: pre-reform (2006) and post-reform (2009, 2011), so as to highlight reform impacts. Similarly, in some specifications we add a reform-income interaction term to the right-hand side, to capture the fact that income affects benefits differently before and after the reform.

Many NCMS-related outcomes could be used on the left-hand side, such as use of healthcare services or out-of-pocket payments; indeed such variables are often used in the literature on health insurance. However, such outcomes are inherently reflective of healthcare needs that may vary with income, in which case “inequality cannot be interpreted as inequity” [2]. Using measures of reimbursement allows to eliminate confounding factors.

We use three measures of benefits: a reimbursement dummy (binary variable for having received reimbursement), the amount of reimbursement received, and the rate of reimbursement (reimbursement/total cost). Depending on the explained variable, we restrict the sample to those who sought medical care or to those who received reimbursement, in order to eliminate concerns about (self-) selection processes.^5^ If, among those who sought medical care, individuals from higher income strata are more likely to obtain reimbursement than poorer ones, it may indicate that the breadth of coverage is not well targeted to the needs of the poor. If, among those who received reimbursement, the wealthier tend to receive higher payments or higher reimbursement rates, it suggests that the depth of coverage is not well targeted to the needs of the poor.

### Explained and explanatory variables

Table 2 summarizes all relevant variables, with means and standard deviations. The reimbursement dummy is equal to 1 if an individual received any NCMS reimbursement during a given year. We also compute the amount of reimbursement received in a year expressed in log, and the fraction of costs reimbursed in a year.

**Table 2:** Summary Statistics for Dependent and Independent Variables, pooling years 2004, 2006, 2009 and 2011

Variable	Description	Observations ^a^	Mean	Standard Dev
Dependent variables				
Reimbursement dummy	1 if reimbursement is greater than zero; 0 otherwise	14469	0.149	0.356
Reimbursement amount	The log of net reimbursement (2004 RMB)	14469	0.707	1.832
Reimbursement rate	Ratio of NCMS reimbursement to total healthcare cost	6554	0.177	0.283
Independent variables at the household level				
Non-transfer income	Per-capita Non-government income (2004 RMB)	14148	7.142	1.130
Transfer income	Per capita income from government transfers (non-NCMS) (2004 RMB)	14468	2.518	2.750
Household size	Excludes long-term migrants	14469	5.063	1.954
Debt dummy	1 if household carries debt at the end of the year, 0 if not	14517	0.577	0.494
Farming type	1 if Head relies exclusively on farming, 2 if do not farm, 3 if rely partly on farming, 0 otherwise	14158	1.436	0.911
Ethnicity	1 if Head ethnicity is Han,0 otherwise	14469	0.641	0.480
Independent variables at the individual level				
Self-reported Health Status	1 if great, 2 if good, 3 if poor, 4 if very poor	14469	2.343	1.319
Member of NCMS	1 if yes, 0 if not	11089	0.86	0.35
Sex	1 if male, 0 if female	14469	0.529	0.499
Age	1 age < 10, 2 if 10 < =age < 40, 3 if age > =40	14469	2.139	0.681
Education	Education type: 1 if primary or less; 2 if middle school; 3 if senior school or above	14469	1.337	0.579
Marriage	1 if single, 2 if married,3 if divorced or other	14469	1.469	0.585
Diagnostic place ^b^	1 if village or town hospital; 2 if county or provincial hospital; 3 if others.	7017	2.172	0.857
Treatment type ^b^	1 if self-treatment; 2 if other informal,3 if saw formal doctor	7793	2.408	0.806
Chronic disease ^c^	1 if individual reports a chronic disease, 0 if not	14469	0.134	0.341

Puding Panel dataset. ^a^: Summing all 4 years of data ^b^: Refers to most significant instance within survey year ^c^: Includes Cardio-vascular diseases, Diabetes, High blood pressure, Arthritis, Hepatitis, Gastric ulcer, Deformity, Lumbar disk degeneration, or others as assessed by respondent

The key independent variable is the natural log of non-transfer income (no household reported zero income). Total non-transfer income was computed using exhaustive information on all sources of income: agricultural production and/or sales, salaries, wage work and odd jobs, gifts and remittances, income from rents, profits from trade, service provision, and non-farm enterprises of all sorts.

Computing incomes from agriculture required calculating the value of output and netting out all costs. Output outliers were identified and corrected on the basis of yield: where recorded output-per-hectare was unrealistically high, the average yield in the village was used to compute a new output estimate. Own consumption of agricultural output was valued at farmgate prices, which were culled from sales data. Cost data collected in the questionnaire was exhaustive and highly disaggregated, including totals spent on seed, fertilizer and other chemicals, irrigation, labor, and mechanized inputs and fuel. Any costs incurred in kind (for instance, seed borrowed from neighbors) was also valued in the questionnaire and therefore easy to incorporate into our calculations. Costs of livestock breeding activities included feed, medicine, labor, veterinary services, and costs of machinery and fuel. Enterprise profits are computed as revenues net of costs, both of which are reported by the respondents for each activity they engage in. Summing income from all these sources yields total non-transfer income. Income coming from government transfers is included in the regressions separately, as it could possibly be an indicator of poverty rather than wealth, since government transfers are often targeted at those who need them most. This is done as a precaution and helps avoid confusion in the interpretation of results, but does not significantly alter them. Transfer income is also expressed in natural log (with 1 RMB added to ensure no missing values).

Running specifications at the individual level allows us to control for self-reported health status (coded on a 1-to-4 scale), sex, age, education and marital status. We also include indicator variables for usual diagnostic place and type of treatment, and, in some specifications, a dummy for NCMS membership. We include household-level controls for farmer households, ethnic minority households, and household size. Some specifications also include a dummy for chronic diseases, and for weather the household was in debt at the end of the previous year.

### Model Specifications

Depending on which variable is on the left-hand side, we run the regressions as probit models, as ordinary least squares (OLS), or as tobit models. Probit regression is appropriate when the explained variable is binary, such as our reimbursement indicator. Probit regression coefficients can be interpreted as impacts on the probability to receive reimbursement. For continuous variables such as reimbursement amounts or reimbursement rates, we can use OLS regressions. To eliminate confounding factors, we run OLS only on the subsample of households who received NCMS reimbursement. This leads to a relatively small sample size in 2006 (*n* = 246 in 2006, *n* = 1792 post-reform). An alternative way of controlling for confounding factors without restricting the sample is to use tobit specifications [39]. This assumes that the distribution of reimbursements is left-censored at zero, and allows for the underlying process governing the amount of reimbursement to be different from the one determining whether any reimbursement was actually received. We run tobits on the larger sample of all those who had medical expenses (1720 observations in 2006). All data compilation and analysis was conducted using the Stata software, regressions made use of the *reg, probit* and *tobit* commands native to the software.

## Results

### Equity in obtaining reimbursement

Table 3 documents the relationship between income levels and the probability of getting reimbursement under NCMS with probit regressions, on the full sample of treatment-seekers. Colum P1 reports the basic probit regression using all three years when NCMS was available (2006, 2009 and 2011). In this specification, the coefficient on our income variable of interest (0.031) is positive and significant at the 10 % level. Higher transfer income (from other public programs) is associated with higher probability of receiving reimbursement under NCMS, possibly indicating that some households are better informed about public transfer opportunities in general, or better able to take advantage of them. The 2009 and 2011 dummies are significant, which suggests that overall reimbursement was easier after the reform. The specification controls for NCMS membership (which is positive and significant), to ensure that we are not observing an enrollment effect.^6^ Poor self-reported health status (3 and 4) also increases the probability of getting reimbursement, even though we restricted the sample to those who received medical attention. This may reflect the fact that those with poor health sought medical attention more often during the year, thus increasing the chance that they will receive reimbursement at one of those occasions at least.

**Table 3:** Income and the probability of getting reimbursement

Dependent Variable: Reimbursement Dummy (1 = yes, 0 = no)				
Probit models	P1	P2	P3	P4
	Three waves with NCMS	Pre-reform	Post-Reform	Three waves, with interaction terms
Years	2006, 2009, 2011	2006	2009, 2011	2006, 2009, 2011
Non-transfer income (ln)^a^	0.031^e^	0.150^c^	0.018	0.144^c^
	−1.831	−3.364	−1.009	−3.556
(Reform dummy) x (Non-transfer income) interaction term^a^				−0.135^c^
				(−3.091)
Transfer income^a^	0.021^d^	0.077^c^	0.015	0.021^d^
	−2.344	−2.894	−1.483	−2.312
2009 dummy	1.153^c^		.	2.096^c^
	−18.436		.	−6.699
2011 dummy	0.832^c^		−0.285^c^	1.777^c^
	−11.879		(−6.115)	−5.641
NCMS member dummy	0.794^c^	1.937^c^	0.470^c^	0.800^c^
	−8.033	−4.988	−3.725	−8.051
SRHS = 2 dummy	0.012	0.006	0.01	0.015
	−0.237	−0.044	−0.18	−0.311
SRHS = 3 dummy	0.240^c^	0.389^c^	0.213^c^	0.244^c^
	−4.51	−2.827	−3.659	−4.583
SRHS = 4 dummy	0.348^c^	0.126	0.929^c^	0.281^c^
	−4.082	−1.033	−2.957	−3.2
N	5461	1720	3741	5461
Chi-squared ^b^	1187.029	228.64	434.017	1196.728
P-stat ^b^	0.000	0.000	0.000	0.000

Puding panel data excluding 2004 (pre-NCMS). Sample restricted to those who sought treatment. All specifications include controls for sex, age, education, marital status, minority status, farmer status, household size, village, place of diagnostic and type of treatment used this year. SRHS = self-reported health status, see Table 2. ^a^: Transfer refers to income received under any local or national governmental programs other than NCMS. ^b^: Overall model fit statistics. ^c, d, e:^ Significantly different from zero at the 0.01, 0.05 and 0.1 level, respectively

Column P1 thus suggests that higher-income households have a better chance of obtaining PSNP reimbursement when they need it, which questions the pro-poor nature of the policy. However, a more subtle picture appears when we run the probit regressions separately for the pre-reform year (2009) and the post-reform years (2009 and 2011). The 2006 probit (P2) displays a positive and strongly significant coefficient on income, but the coefficient in the post-reform probit (2009 and 2011, P3) is not significantly different from zero. This suggests that richer households had an easier time securing reimbursement for their medical expenses in 2006, but not in 2009 and 2011, after the reform. Column P4 uses all treatment-seekers and adds a reform-income interaction term. It shows income is positively associated with a probability of reimbursement (0.144, significant at the 1 % level), but most of this disappears in the reform years (-0.135, also significant at the 1 % level). It appears NCMS reforms changed the relationship between income and access to reimbursement.

### Equity in reimbursement amounts and rates

Table 4 reports regressions of the amount of reimbursement received under NCMS by an individual during the year. We report three ordinary least-squares regressions (OLS) using the sample of those who received reimbursement under NCMS, and three tobit regressions on the sample of treatment-seekers, using the same control variables as in Table 3. We run OLS specifications. Columns R1 and R2 respectively restrict the sample to pre-reform (2006) and post-reform (2009 and 2011). Column R3 pools the three NCMS years of data, and includes the reform-income interaction term. The coefficients on income in columns R1 and R2 are of opposite signs: positive before the reform (0.138, not significant), negative after (−0.056, significant at 5 %). Column R3 confirms those signs, with a positive and significant sign on the log of income, but a negative and significant coefficient on the interaction term. These signs are consistent with a scenario where richer individuals tended to receive higher NCMS reimbursements in 2006, but smaller payments after the reform. This suggests that the reform may have made NCMS more progressive.

**Table 4:** Income and the amount of reimbursement

Dependent Variable: Ln of reimbursement amount received (2004 RMB)						
Model	Ordinary least squares			Tobit		
Specification	R1	R2	R3	T1	T2	T3
	Pre-reform	Post-Reform	Three waves, with interaction terms	Pre-reform	Post-Reform	Three waves, with interaction terms
	2006	2009, 2011	2006, 2009, 2011	2006	2009, 2011	2006, 2009, 2011
Non-transfer income (ln)^a^	0.138	−0.056^d^	0.254^c^	0.929^c^	0.03	0.750^c^
	−1.351	(−1.987)	−2.596	−3.483	−0.42	−4.143
(Reform dummy) x (Non-transfer income)^a^			−0.316^c^			−0.764^c^
			(−3.123)			(−3.948)
Transfer income^a^	0.008	−0.005	−0.013	0.442^c^	0.057	0.083^d^
	−0.14	(−0.345)	(−0.882)	−2.789	−1.474	−2.189
2009 dummy		.	2.732^c^		.	10.708^c^
		.	−3.723		.	−7.695
2011 dummy		0.464^c^	3.217^c^		−0.845^c^	9.691^c^
		−6.01	−4.371		(−4.564)	−6.928
NCMS member dummy				11.965^c^	1.864^c^	3.647^c^
				−5.15	−3.658	−8.397
SRHS = 2 dummy	0.373	0.124	0.134	0.218	0.108	0.15
	−1.149	−1.446	−1.615	−0.276	−0.508	−0.721
SRHS = 3 dummy	0.663^d^	0.741^c^	0.727^c^	2.595^c^	1.313^c^	1.483^c^
	−2.141	−8.306	−8.478	−3.192	−5.808	−6.706
SRHS = 4 dummy	−0.683^d^	0.628	−0.098	0.466	3.644^c^	1.332^c^
	(−2.464)	−1.609	(−0.549)	−0.644	−3.304	−3.438
N	246	1792	2038	1720	3741	5461
R-squared^b^	0.440	0.294	0.300			
Chi-squared^b^				253.03	550.57	1392.6
P-stat^b^	0.000	0.000	0.000	0.000	0.000	0.000

Puding panel data excluding 2004 (pre-NCMS). R1, R2, R3: sample restricted to those who received reimbursement; T1, T2, T3: sample restricted to those who sought treatment. All specifications include controls for sex, age, education, marital status, minority status, farmer status, household size, village, place of diagnostic and type of treatment used this year. SRHS = self-reported health status, see Table 2. ^a^: Transfer refers to income received under any local or national governmental programs other than NCMS. ^b^: Overall model fit statistics. ^c, d,^ ^e^: Significantly different from zero at the 0.01, 0.05 and 0.1 level, respectively

Columns T1, T2 and T3 repeat the same respective specifications as R1 R2 and R3, using tobit instead of OLS, and the larger sample. Specification T1 shows a positive and strongly significant coefficient on income (0.929), but in specification T2 this coefficient is very small and insignificant (0.03). Column T3 bolsters significance across all coefficients of interest.

It may be that lower-income villagers receive smaller reimbursement amounts (in 2006) because they use the medical system less intensively than richer ones. To verify this, we run all the same specifications again, but with the rate of reimbursement (reimbursement/cost) as the explained variable. Results are presented in Table 5. The results are very similar to those of Table 4 in terms of significance of coefficients. In 2006, the rich were getting a greater share of their medical expenditures reimbursed, but not after the reform. Both 2009 and 2011 year dummies appear significantly positive, consistent with the fact that the reform increased the reimbursement rate for many diseases.

**Table 5:** Income and the rate of reimbursement (restricted samples)

Dependent Variable: Rate of reimbursement received						
Model	Ordinary least squares			Tobit		
	R1’	R2’	R3’	T1’	T2’	T3’
	Pre-reform	Post-Reform	Three waves, with interaction terms	Pre-reform	Post-Reform	Three waves, with interaction terms
	2006	2009, 2011	2006, 2009, 2011	2006	2009, 2011	2006, 2009, 2011
Non-transfer income (ln)^a^	0.034^e^	−0.005	0.032^d^	0.107^c^	0.006	0.090^c^
	−1.856	(−1.341)	−2.329	−3.769	−0.663	−4.177
(Reform dummy) x (Non-transfer income)^a^			−0.036^c^			−0.088^c^
			(−2.600)			(−3.860)
Transfer income^a^	−0.007	0.004^d^	0.003^e^	0.045^c^	0.010^d^	0.013^c^
	(−0.687)	−2.135	−1.69	−2.689	−2.188	−2.829
2009 dummy		.	0.405^c^		.	1.280^c^
		.	−3.978		.	−7.778
2011 dummy		−0.020^e^	0.386^c^		−0.144^c^	1.117^c^
		(−1.937)	−3.78		(−6.513)	−6.753
NCMS member dummy				1.255^c^	0.219^c^	0.409^c^
				−4.965	−3.596	−8.045
SRHS = 2 dummy	0.02	0.001	0.002	0	0.004	0.007
	−0.34	−0.081	−0.179	−0.002	−0.162	−0.288
SRHS = 3 dummy	−0.013	0.008	0.005	0.221^d^	0.102^c^	0.118^c^
	(−0.233)	−0.644	−0.393	−2.553	−3.748	−4.548
SRHS = 4 dummy	−0.077	0.056	−0.048^e^	0.046	0.428^c^	0.136^c^
	(−1.542)	−1.07	(−1.957)	−0.596	−3.247	−2.987
N	246	1792	2038	1720	3741	5461
R-squared^b^	0.162	0.025	0.095			
Chi-squared^b^				215.942	435.247	1316.162
P-stat^b^	0.012	0.007	0.000	0.000	0.000	0.000

Puding panel data excluding 2004 (pre-NCMS). R1’, R2’, R3’: sample restricted to those who received reimbursement; T1’, T2’, T3’: sample restricted to those who sought treatment. All specifications include controls for sex, age, education, marital status, minority status, farmer status, household size, village, place of diagnostic and type of treatment used this year. SRHS = self-reported health status, see Table 2. ^a^: Transfer refers to income received under any local or national governmental programs other than NCMS. ^b^: Overall model fit statistics. ^c, d, e^: Significantly different from zero at the 0.01, 0.05 and 0.1 level, respectively

### Exploring impact channels

Several mechanisms may be underlying the relationship between incomes and reimbursements. We already control for usual place of diagnostic and treatment type, so those are not likely to be the main drivers of what we observe. Two of the main clauses of the 2009 reforms were the reimbursement of outpatient care, and the implementation of a simultaneous reimbursement system. Both of these provide impact channels that could explain our results, and our data can provide partial clues as to whether those mechanisms are at work here.

The poor and the wealthy may be vulnerable to (or seek treatment for) different types of diseases, which may be covered differently by the insurance. If lower-income people were disproportionately in need of outpatient care, the 2009 reform would allow them to obtain more reimbursement, more often, consistently with our results. Although we do not have detailed information on health conditions of individuals, we do know whether or not they suffer from a chronic disease. Since the treatment of many chronic diseases requires outpatient services rather than hospitalization, controlling for chronic disease may pick up some of this effect. Chronic diseases were reported by 13 % of the sample.

Cash constraints could also explain our results. Even if they expect reimbursement, cash constrained patients may be unwilling or unable to disburse the insured share of costs. Simultaneous reimbursement alleviates the need for cash upfront. We can use indebtedness to proxy for how cash-constrained a household is. Over half of the sample reported carrying debt at the end the year.

Table 6 presents regression results using the same specifications as presented so far, with two additional dummy variables: an indicator for whether the individual suffers from chronic illnesses, and another for whether their household carried outstanding debt at the end of the previous year. We use the lagged debt variable because current-year debt may be resulting from medical expenditures, which would confuse the interpretation of coefficients.^7^

**Table 6:** Specifications including controls for chronic diseases and cash constraints

	(1)	(2)	(3)	(4)	(5)	(6)	(7)	(8)	(9)	(10)
Model	Probit	Probit	Ols	Ols	Tobit	Tobit	Ols	Ols	Tobit	Tobit
Dependent variable: Reimbursement […]	Dummy	Dummy	Amount (ln)	Amount (ln)	Amount (ln)	Amount (ln)	Rate	Rate	Rate	Rate
Period	Pre-reform	Post-reform	Pre-reform	Post-reform	Pre-reform	Post-reform	Pre-reform	Post-reform	Pre-reform	Post-reform
Years	2006	2009,2011	2006	2009,2011	2006	2009,2011	2006	2009,2011	2006	2009,2011
Non-transfer income (ln) ^a^	0.170^c^	−0.003	0.169	−0.056^e^	1.063^c^	−0.053	0.038^e^	−0.004	0.118^c^	−0.003
	−3.504	(−0.157)	−1.545	(−1.834)	−3.682	(−0.660)	−1.964	(−0.883)	−4	(−0.355)
Transfer income^a^	0.074^c^	0.018	−0.013	−0.003	0.407^d^	0.066	−0.006	0.005^d^	0.041^d^	0.011^d^
	−2.66	−1.608	(−0.227)	(−0.194)	−2.496	−1.499	(−0.597)	−2.12	−2.504	−2.168
Chronic disease	−0.205^e^	0.191^c^	0.415^e^	0.569^c^	−1.035	1.162^c^	−0.02	−0.004	−0.130^e^	0.089^c^
	(−1.757)	−2.98	−1.74	−5.708	(−1.532)	−4.585	(−0.461)	(−0.288)	(−1.869)	−2.944
Debt from last year	−0.006	−0.147^c^	−0.286	−0.117	−0.045	−0.660^c^	−0.014	0.006	−0.003	−0.065^c^
	(−0.063)	(−2.893)	(−1.405)	(−1.501)	(−0.084)	(−3.293)	(−0.375)	−0.609	(−0.057)	(−2.697)
NCMS member dummy	1.879^c^	0.612^c^			11.459^c^	2.523^c^			1.163^c^	0.311^c^
	−4.74	−4.082			−4.88	−4.07			−4.763	−4.188
2011 dummy		−0.279^c^		0.449^c^		−0.831^c^		−0.012		−0.138^c^
		(−5.520)		−5.565		(−4.113)		(−1.097)		(−5.719)
SRHS = 2 dummy	0.043	0.019	0.356	0.111	0.39	0.104	0.007	0.006	0.021	0.01
	−0.306	−0.311	−1.094	−1.176	−0.471	−0.436	−0.115	−0.479	−0.253	−0.334
SRHS = 3 dummy	0.458^c^	0.158^d^	0.703^d^	0.588^c^	2.940^c^	0.983^c^	−0.034	0.015	0.244^c^	0.080^d^
	−3.029	−2.367	−2.164	−5.876	−3.34	−3.764	(−0.583)	−1.126	−2.711	−2.548
SRHS = 4 dummy	0.058	0.788^d^	−0.476^e^	0.806^e^	0.115	3.484^c^	−0.109^d^	0.063	−0.006	0.407^c^
	−0.449	−2.267	(−1.664)	−1.716	−0.153	−2.652	(−2.137)	−0.995	(−0.080)	−2.598
N	1467.000	3159.000	226.000	1495.000	1467.000	3159.000	226.000	1495.000	1467.000	3159.000
Chi-squared ^b^	202.971	359.521			226.073	467.421			192.599	356.474
R-squared ^b^			0.472	0.334			0.178	0.024		
P-stat ^b^	0.000	0.000	0.000	0.000	0.000	0.000	0.020	0.082	0.000	0.000

Puding panel data. OLS: sample restricted to those who received reimbursement; Probit, Tobit: sample restricted to those who sought treatment. All specifications include controls for sex, age, education, marital status, minority status, farmer status, household size, village, place of diagnostic and type of treatment used this year. SRHS = self-reported health status, see Table 2. ^a^: Transfer refers to income received under any local or national governmental programs other than NCMS. ^b^: Overall model fit statistics. ^c, d, e^: Significantly different from zero at the 0.01, 0.05 and 0.1 level, respectively

Results show that the reform made a significant difference for people with chronic diseases. Having a chronic disease made people 20 % less likely to obtain reimbursement before 2006, but 19 % more likely after the reform (columns 1 and 2). The tobit specifications suggest that the NCMS only increases the amounts reimbursed after the reform (significant 1.162 in column 6, but negative insignificant in column 5). If we restrict the sample to those who received reimbursement in 2006 (and thus were hospitalized), a chronic disease does increase the reimbursement amounts, which may be reflecting the severity of their condition (column 3). However, the coefficient becomes larger and more significant after the reform (column 4). Columns 7 and 8 show no significant impact, but columns 9 and 10 suggest that patients with chronic diseases received significantly lower reimbursement rates before the reform (−0.13), but higher rates thereafter (0.089).

The lagged debt variable appears negative in all specifications, and significant in some (columns 2, 6 and 10). The negative sign confirms that cash-constrained households find it harder to benefit from NCMS. Debt makes patients who sought treatment less likely to receive reimbursement. Debtors who do receive reimbursement get smaller amounts, and lower reimbursement rates. This may seem surprising, since cash constraints should incentivize patients to seek reimbursable treatments. However, this could also reflect the fact that cash constrained households simply do not seek care for expensive conditions. The fact that coefficients on debt are larger and more significant after the reform suggests that “simultaneous reimbursement” has not fully solved the issue of cash constraints and healthcare.

### Discussion

With respect to our analysis of NCMS, the uniqueness of this dataset stems from its panel nature and the information it gathers about health expenditures and reimbursements. A majority of studies of NCMS rely on cross-section data or 2-year panels. In contrast our data offers three waves of panel and span 5 years of NCMS existence, with a major reform in that period. This allows us to look beyond impacts of NCMS existence itself and start honing in on specific provisions of the policy. In addition, this dataset is a complete census, with data on every single household in the area, which avoids all issues related to sampling. Health information is recorded for every member of the household, which is another important feature. This provides us with a unique opportunity to study the mechanisms which determine the pro-poor nature of a health insurance scheme.

The drawback of having such high quality data is the limited representativeness. Our sample is only representative of a small area, and we make no claims to external validity. In an ideal world, we would have such data for the entire country, rather than a group of villages, enabling us to evaluate NCMS nationally. However, such data is not available and cannot be collected *ex-post*. Other available surveys tend to have other issues, related to the representativeness of rural areas (NCMS is only a rural program), panel continuity, information at the member level etc. In addition, NCMS implementation and reforms differ somewhat among provinces, which complicates evaluation at the higher scale. For our purposes, a perfectly representative dataset for a small region is a more effective choice.

Continued analysis of panel datasets such as the one we use has the potential to elucidate the conditions under which health insurance policies can reach their equity goals. Our results only partially answer this question, and should be interpreted with caution. A cause of concern is that we cannot assess the degree to which epidemiology plays a role in generating our results: post-reform years could appear different because of the particular illnesses that were prevalent in those years, not because of the reform itself. Medical records would be necessary to rule out such explanations. In general, clinical information would help us explain why lower-income households have more equal access to reimbursements after the reform. A well-designed pro-poor insurance scheme should arguably never favor higher incomes regardless of shifts in disease patterns.

## Conclusions

Reducing medical impoverishment and inequality are part of the stated goals of the NCMS. Finding out whether a policy is pro-poor presents many challenges, and understanding how it is meeting those goals is even more challenging. We are able to provide some answers by using a unique dataset from Puding county, and taking advantage of program reforms. Our results suggest that while the early version of NCMS favored higher-income households in our sample, the program became more progressive after the 2009 reform. Such results are highly relevant to the debate over health insurance.

A widely held view argues that the poor need to be protected against medical impoverishment triggered by catastrophic illnesses or accidents. In that spirit, NCMS started as a program that covered hospitalizations. However, our results suggest that, in our sample, NCMS favored higher incomes until it allowed for the reimbursement of outpatient services. We also show that the reform likely answered a significant pent-up demand for insurance of chronic conditions. The most essential insurance needs of the poor lie perhaps not in catastrophic illnesses, but rather in the small and regular medical expenditures.

Our results also speak to the issue of cash constraints and access to medical services. The 2009 reforms implemented simultaneous reimbursement, partly alleviating the financial burden associated with medical care. While the post-reform NCMS reimbursements appear unrelated to income in our results, households that are in debt still fail to benefit as much as those who are not. More reforms may be necessary to create a scheme which does not disadvantage the cash-poor.

Next steps in this research must further disentangle the different aspects of the NCMS rules and reforms (breadth of coverage, depth of coverage, etc.), to figure out which particular provisions are most crucial to the progressive nature of the policy. Longer panel datasets and detailed medical records will likely be necessary to deepen our understanding of the results we present in this paper.

Inequality in access to medical care is a serious issue common to all countries. Beyond the obvious implications for China’s government, this research has high relevance in the debate over publicly-sponsored health insurance. Understanding how to make health insurance pro-poor may be one of the keys to dismantling the vicious circle of poverty and ill-health.

## Abbreviations

NCMS: New cooperative medical scheme
OLS: Ordinary least squares

## Footnotes

1 The “New” Cooperative Medical Scheme is related to the “Rural Cooperative Medical Systems” (RCMS) which were encouraged in the 60’s and 70’s but abandoned during market economic reforms (Singer Babiarz et al. 2010). The Chinese government had been working on re-instating a rural insurance scheme since the mid-nineties [40].

2 The data was jointly collected by the International Food Policy Research Institute (IFPRI), the Chinese Academy of Agricultural Sciences (CAAS), and Guizhou University.

3 The official poverty rate may differ from year to year. It was 668 RMB in 2004, about 188 USD in PPP terms (using the 3.55 private consumption conversion factor published by the World Bank, 2015). It was raised to 693 RMB(2006), 1196 RMB (2009) and 2300 RMB (2011), reflecting not only inflation but also changes in definition. We use the 2004 poverty line to ensure comparability.

4 It is interesting to note that the participation rate, while very high, decreased by three percentage points between 2009 and 2011. Unfortunately, we have no way of knowing why this 3 % failed to renew their enrollment. Plausible explanations could be that they did not want to pay the 30 Yuan fee, that they did not know they needed to renew membership, or that they were procrastinating*.* It could also be that the enrollment drive was less active in 2011. Regardless, at these levels of enrollment we are not too worried about selection bias driving our results. (We still control for enrollment as a precaution).

5 The decisions to seek medical care or to become a member of NCMS raise bias and endogeneity questions which are not our concern here.

6 Outcomes are almost identical if we restrict to treatment-seekers enrolled in NCMS, to all enrolled in NCMS, or use the full sample, suggesting that self-selection mechanisms are not a major force at play in our sample.

7 Previous-year and current-year debt variables all yield similar results. The results are also robust to using the amount of debt owed or the ratio of indebtedness in place of a debt dummy.
